# Diet influences the functions of the human intestinal microbiome

**DOI:** 10.1038/s41598-020-61192-y

**Published:** 2020-03-06

**Authors:** Maria De Angelis, Ilario Ferrocino, Francesco Maria Calabrese, Francesca De Filippis, Noemi Cavallo, Sonya Siragusa, Simone Rampelli, Raffaella Di Cagno, Kalliopi Rantsiou, Lucia Vannini, Nicoletta Pellegrini, Camilla Lazzi, Silvia Turroni, Nicola Lorusso, Mario Ventura, Marcello Chieppa, Erasmo Neviani, Patrizia Brigidi, Paul W. O’Toole, Danilo Ercolini, Marco Gobbetti, Luca Cocolin

**Affiliations:** 1 Department of Soil, Plant and Food Science, University of Bari Aldo Moro, Bari, Italy; 2 Department of Agricultural, Forest and Food Science, University of Turin, Grugliasco, Italy; 3 Department of Biology, University of Bari Aldo Moro, Bari, Italy; 4 Department of Agricultural Sciences and Task Force on Microbiome Studies, University of Naples Federico II, Portici, Italy; 5 Department of Pharmacy and Biotechnology, Alma Mater Studiorum University of Bologna, Bologna, Italy; 6 Faculty of Science and Technology, Free University of Bozen, Bozen, Italy; 7 Department of Agricultural and Food Sciences, Alma Mater Studiorum University of Bologna, and Inter-Departmental Centre for Industrial Agri-Food Research, Alma Mater Studiorum University of Bologna, Cesena, Italy; 8 Food and Drug Department, University of Parma, Parma, Italy; 9 National Institute of Gastroenterology “S. de Bellis”, Castellana Grotte, Bari, Italy; 10 Department of Microbiology and Alimentary Pharmabiotic Centre, University College Cork, Cork, Ireland

**Keywords:** Metagenomics, Microbiome

## Abstract

Gut microbes programme their metabolism to suit intestinal conditions and convert dietary components into a panel of small molecules that ultimately affect host physiology. To unveil what is behind the effects of key dietary components on microbial functions and the way they modulate host–microbe interaction, we used for the first time a multi-omic approach that goes behind the mere gut phylogenetic composition and provides an overall picture of the functional repertoire in 27 fecal samples from omnivorous, vegan and vegetarian volunteers. Based on our data, vegan and vegetarian diets were associated to the highest abundance of microbial genes/proteins responsible for cell motility, carbohydrate- and protein-hydrolyzing enzymes, transport systems and the synthesis of essential amino acids and vitamins. A positive correlation was observed when intake of fiber and the relative fecal abundance of flagellin were compared. Microbial cells and flagellin extracted from fecal samples of 61 healthy donors modulated the viability of the human (HT29) colon carcinoma cells and the host response through the stimulation of the expression of Toll-like receptor 5, lectin RegIIIα and three interleukins (IL-8, IL-22 and IL-23). Our findings concretize a further and relevant milestone on how the diet may prevent/mitigate disease risk.

## Introduction

Comprising 10^12-14^ cells from 100–1000 species, the human intestinal microbiome is a complex entity living in our body. It affects human health, sustenance and well-being^1^. Functioning as an extra organ^2^, the intestinal microbiome uses nutrients from ingested foods, releases harmful or beneficial metabolites and regulates the immune system^3,4^. Dysbiosis of the intestinal microbiome has been associated to various gastrointestinal (GI) and non-GI diseases, such as obesity, heart-, kidney- and liver-related diseases, cancer and autism^5–7^, but the dilemma whether it acts as cause or consequence remains too far from the solution. Because the simple phylogenetic characterization does not provide deep information on functional repertoires^8,9^, a number of projects and studies have employed the whole-community shotgun sequencing to combine the composition and gene content of the human intestinal microbiome^2,10^. Current meta-genomic data show limited differences among individuals, which hypothesize the existence of a stable core microbiome and similar metabolic traits^10^. Notwithstanding the relevance of these findings, they do not necessarily imply similar *in vivo* microbial activities. Indeed, a panel of factors (e.g., diet, host genetics and pharmacological treatments) markedly affect such activities^11^. Dietary components have the capability to modulate the composition and mainly the function of the intestinal microbiome^3,12,13^. Correlations between entero-gradients/types and vegetable-rich (*Prevotella* and *Lachnospira*) or animal protein-rich diets (*Bacteroides* and Clostridia/*Ruminococcus*) have been already highlighted^12,14^, but the impact of dietary components still remains unclear. Previously, we have determined the compositional structure of the fecal microbiota and metabolome of 150 healthy omnivorous, vegan and vegetarian volunteers, demonstrating that vegetable-rich foods increased both the abundance of fiber-degrading bacteria and the synthesis fecal short-chain fatty acids (SCFAs)^14^. In contrast, omnivorous volunteers having low adherence to the Mediterranean diet (MD) had the highest levels of detrimental microbial metabolites, such as phenolic and indole derivatives, and trimethylamine N-oxide (TMAO). This emerging picture hypothesizes that diet modulates the functionality of the intestinal microbiome, which, in turn, affects the human metabolic status^15,16^.

In this study, we implemented a multi-omic approach (meta-genomic, -proteomic and -metabolomics) to thoroughly characterize fecal samples from omnivorous, vegan and vegetarian volunteers with the aim of showing the molecular relationship between diet and metabolic functions of the intestinal microbiome. We sought to identify the effects of key dietary components on microbial functions that modulate host-microbe interactions. This approach might lead to effective intervention strategies for maintaining human health via the diet-microbiome axis.

## Results

### Fecal microbiome composition

Thirty volunteers were selected from a previous larger cohort^14^ where healthy adult non-smokers (15 men and 15 women) were enrolled, with an age of 25–55 years (36 ± 7.0), and a BMI > 18 (21.89 ± 2.20). All volunteers were on omnivorous, vegetarian or vegan diet for at least one year. Supplementary Table S1 in the online supplemental material describes the diet compositions together with the comparison of dietary intakes among the three considered diet groups (analysis of variance (ANOVA)). The degree of association between samples and dietary information as measured by Spearman’s correlations, (rows and columns are clustered by average-linkage clustering) allowed us to cluster omnivorous, vegetarian and vegan volunteers (Supplementary Fig. S1). Each individual collected fecal samples at three time-points (within one month), and the three samples were pooled. According to our previous findings^14^, the fecal microbiome of the individuals differed slightly (data not shown). *Lachnospira* was significantly (FDR < 0.05) associated to vegans and vegetarians, while *Ruminococcaceae* was the most abundant family for omnivores.

### Fecal microbiome gene catalogue

To establish associations between microbiome genes and omnivorous, vegan or vegetarian diet, we developed a comprehensive meta-genome catalogue performing shotgun sequencing of the total DNA from fecal specimens. The poor DNA quality excluded three samples (two omnivores and one vegetarian). The remaining 27 samples gave 184 Gb sequences, with an average of 6.81 Gb per sample. Reads assembled into 1.68 M contigs longer than 500 bp. The total contig length per sample was 96 Mb with an N50 length of 2.3 kb (Supplementary Table S2).

The functional characterization and gene classification of the shotgun sequence reads was performed against the Integrated Gene Catalogue (IGC)^17^ database and the MetaHIT gene catalogue, inclusive of close-to-complete sets of genes for most gut microbes. Specifically, the MetaHIT project collected more than 1200 sequenced metagenomics samples of the Human Intestinal Tract (see materials and methods section). Based on this resource, we detected 3,644 KEGG Orthology (KO) genes, with 90% of samples sharing 2,227 of them. MetaPhlAn2^18^ was used to determine the phylogenetic abundances; the related estimated α-diversity indices revealed how intestinal microbiomes were not discriminated by diet. No significant differences were detectable at the phylum level (data not shown), but several genera associated to the intake of specific dietary components. For instance, *Lachnospira* positively correlated to the intake of beta-carotene, vitamin E and vegetable fat but negatively with meat, proteins, cholesterol and total proteins (Supplementary Fig. S2).

### Meta-genomes associated to diets

**Figure 1:**
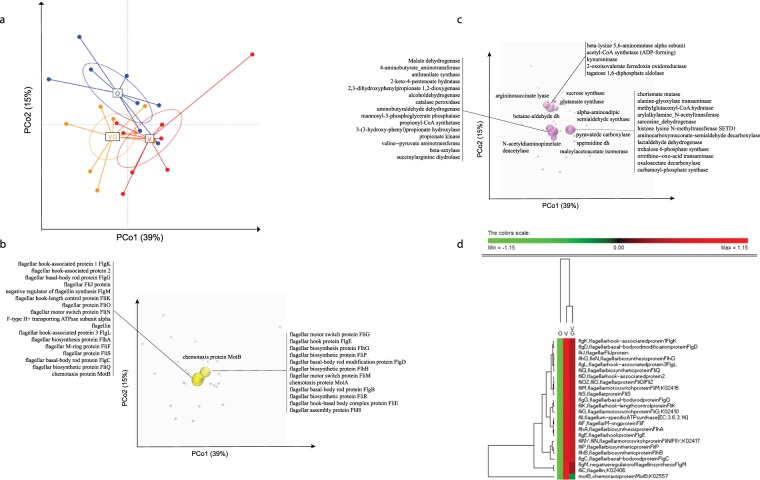
Principal coordinate analysis (PCoA) of the KEGG Orthology (KO) genes that discriminated the fecal microbiomes of omnivorous (O), vegan (V) and vegetarian (VG) volunteers based on their diets. Panel a, Euclidean PCoA plot illustrating the observed diversity between samples. Panel b, the most abundant KO genes belonging to carbohydrate and amino acid metabolism. Panel c, the most abundant KO genes involved in flagellar assembly and bacterial chemotaxis. The spheres represent KO genes mapped onto the weighted average of the coordinates of all samples, where the weights are the relative abundances of the genes in the samples. The size of the spheres is proportional to the mean relative abundance of the corresponding genes across all samples. Purple spheres represent amino acid or carbohydrate metabolism; yellow spheres represent flagellar assembly, bacterial chemotaxis or two-component system genes. Panel d, heatmap showing the differentially (FDR < 0.05) detected genes involved in flagellar assembly and bacterial chemotaxis. The colors of the scale bar denote the abundance of the genes, with 1.15 indicating the highest abundance (red) and −1.15 indicating the lowest abundance (green) between diet groups.

Based on principal coordinate analysis (PCoA) using relative gene abundance, microbiomes grouped into three different clusters corresponding to diets (Fig. 1a). As defined by permutation multivariate analysis of variance (PERMANOVA) test, each cluster showed different microbiome layouts. Then, we applied Random Forests^19^ to functional data set. This allowed the identification of diet discriminatory KO genes having distinctive changes in abundance (Fig. 1b,c and Supplementary Table S3). Superimposing the biplot of KO gene coordinates on the PCoA plot, we identified those genes that differed among omnivores, vegans and vegetarians. Genes responsible for amino acid and carbohydrate metabolisms (Fig. 1c and Supplementary Table S3), two-component gene regulatory system (Ko02020), chemotaxis (Ko02030), and, especially, flagellar assembly (Ko02040) were associated to vegan and/or vegetarian diets (Fig. 1c,d and Supplementary Table S3). We used the Bioconductor package DESeq2^20^ to investigate the relative abundance of specific genes depending on dietary habits. The DESeq2 comparison among the three diet groups evidenced statistically significant gene differences. The highest number of differentially abundant genes (adjusted p-values, calculated with Wald test in DESeq2 followed by Benjamini–Hochberg correction) was found comparing omnivores and vegans (Supplementary Table S4). Discriminatory genes were mainly responsible for cell mobility, environmental information, genetic information processing, and carbohydrate, amino acid, energy, nucleotide, cofactors and vitamins, lipid, and glycan biosynthesis metabolisms.

### Meta-proteomes associated to diets

**Figure 2:**
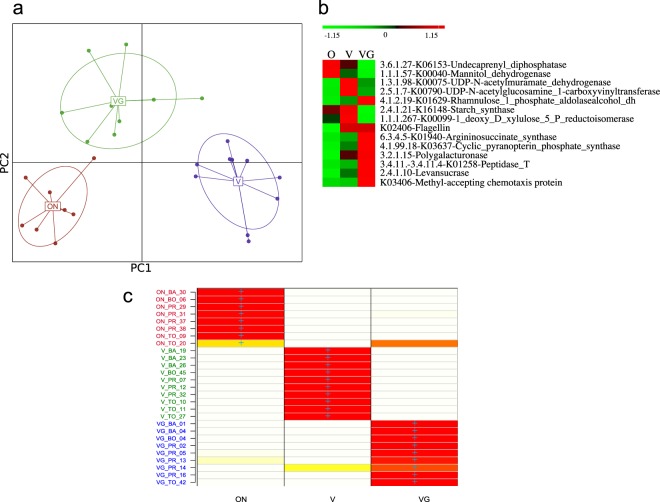
Multivariate statistical analyses based on the meta-proteomes of omnivores (O), vegans (V) or vegetarians (VG). Panel a, discriminant analysis of principal components (DAPC) score plot. Panel b, heatmap showing the differentially (FDR < 0.05) detected proteins in the sample meta-proteomes that mostly discriminated the diet groups. The colors of the scale bar denote the protein abundance with 1.15 indicating the highest abundance (red) and −1.15 indicating the lowest abundance (green) between diet groups. Panel c represents whether the individuals (rows) were correctly assigned (based on discriminant functions) to the genetic cluster where they were included a priori (columns) by K-means analyses used to infer the best-supported clustering solution. Colors represent membership probabilities to each cluster (red = 1, orange = 0.75, yellow = 0.25, white = 0) and blue crosses indicate the cluster where the individuals were originally assigned by K-means analyses. Sample label colours match the sample diet labels in the DAPC clusters (Panel a).

Thanks to a proteomic gel-free method based on ultra-high-pressure liquid chromatography tandem mass spectrometry (UHPLC-MS/MS) performed with Easy-nLC 1000 UHPLC system coupled to a quadrupole-Orbital mass spectrometer, we identified 5,760 proteins with EggNOG function, 2,950 of which associated to a KO code. On average, more than 90% of the proteins belonged to Firmicutes, Bacteroidetes, Actinobacteria and Proteobacteria (data not shown). We used the Bioconductor package DESeq2, PCoA and discriminant analysis of principal components (DAPC) to stratify samples and to identify proteins specific for dietary groups. In vegetable-rich diets, the intestinal microbiome showed statistically significant differences in the synthesis of 181 proteins (Supplementary Table S5), which were mainly responsible for carbohydrate, nitrogen and cofactor and vitamin metabolisms. Other differences related to environmental information processing and genetic information processing (replication and repair). A ribonuclease (K03601, E.C. 3.1.11.6) responsible for DNA replication and repair clearly associated with vegetable-rich diets. PCoA of the core protein relative abundance clustered samples (FDR = 0.040; permutational test with pseudo F-Ratio) depending on the diet (data not shown). DAPC group classification was consistent with the original clusters (prior provided clusters) of omnivores, vegans and vegetarians (Fig. 2a). Flagellin (K02406), cyclic pyranopterin monophosphate synthase, polygalacturonase and levansucrase proteins specifically discriminated vegans and vegetarians from omnivores (Fig. 2b). The omnivorous cluster had the highest relative amount of undecaprenyl diphosphatase. The vegetarian cluster distinguished because of argininosuccinate synthase, cyclic pyranopterin monophosphate synthase, peptidase T and methyl-accepting chemotaxis protein. UDP-N-acetylglucosamine 1-carboxyvinyl transferase, UDP-N-acetylmuramate dehydrogenase, starch synthase and xylulose 5-P-reductoisomerase specifically distinguished the vegan cluster. Based on the retained discriminant functions, the analysis derives probabilities for each individual of membership in each of the different groups. The posterior DAPC assignments were consistent with the original pre set diet clusters (Fig. 2c).

### Diet modulates carbohydrate metabolism and biosynthesis of SCFA of the intestinal microbiome

**Figure 3:**
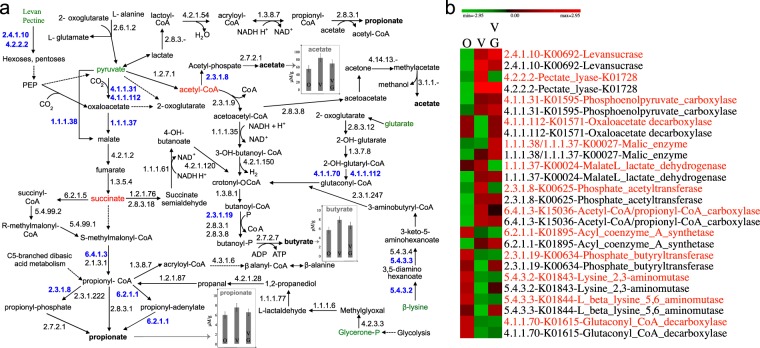
Reconstruction of microbial pathways in the intestine involved in the biosynthesis of short-chain fatty acids (acetic acid, butanoate and propionate) (SCFAs) using DESeq statistically significant differences for genes and proteins identified from the multi-omics data sets belonging to omnivores (O), vegans (V) and vegetarians (VG). Panel a, schematic representation of the SCFA metabolic pathways. The blue numbers indicate enzymes that were differentially (FDR < 0.05) detected among diet groups. Principal metabolites are colored in green. The average concentrations (µM/g of feces) of acetate, butyrate and propionate found in the metabolome of omnivores, vegans and vegetarians are indicated in the histograms. Panel b, heatmap showing the differentially detected genes (red characters) and proteins (black characters) in the diet groups. The colors of the scale bar denote the abundance of the genes and proteins (indicated in blue characters in panel a), with 1.94 indicating the highest abundance (red) and −1.94 indicating the lowest abundance (green) between diet groups.

Biology Pathway Tools (PT) software and the MetaCyc multiorganism database^21^ were used to reconstruct metabolic pathways from meta-genomic and -proteomic data. This innovative approach enabled the reconstruction of the main metabolic pathways, which were active in fecal samples. We compared enzymes at two levels. At the first level, we compared the averaged values (relative amount) of the same gene detected in each diet cohort (omnivores *vs*. vegans, omnivores *vs*. vegetarians and vegans *vs*. vegetarians). At the second level, we compared the averaged values of the same protein detected in each diet cohort. Both meta-genomic and meta-proteomic data showed the synthesis of SCFAs through carbohydrates and free amino acids (Fig. 3a). Fecal samples from vegans and vegetarians harboured the highest levels of genes and/or proteins with statistically significant differences emerging by applying DESeq2 software (e.g., levansucrase, E.C. 2.4.1.10; pectate lyase, E.C. 4.2.2.2; and phosphoenolpyruvate carboxylase, E.C. 4.1.1.31) capable of hydrolyzing acetyl-CoA and succinate (Fig. 3a,b). Acetyl-CoA was used to synthesize acetate by phosphate acetyltransferase (E.C. 2.3.1.8), which was at a higher level in vegetarians and, especially, vegans. The main routes to synthesize butyrate and propionate were acetyl-CoA and succinate pathways, respectively. Vegans and vegetarians also had the highest level of the super-pathway of *Clostridium* acidogenic fermentation, which resulted in the synthesis of butyrate from pyruvate/acetyl-CoA. On the contrary, the metabolic pathway converting amino acids (β-lysine and glutarate) into butyrate mainly associated to omnivores. The levels of acetyl-CoA/propionyl-CoA carboxylase (E.C. 6.4.1.3) and acyl-coenzyme A synthetase (E.C. 6.2.1.1) from the succinate pathway were the highest in vegans and vegetarians. As determined by headspace solid-phase micro-extraction (SPME), coupled with gas chromatography mass spectrometry (GC-MS), a higher concentrations of acetate, butyrate and propionate was found in the fecal samples of vegans and vegetarians with respect to omnivores, which confirmed meta-genomic and -proteomic results (Fig. 3a).

### Diet modulates the nitrogen metabolism of the intestinal microbiome

**Figure 4:**
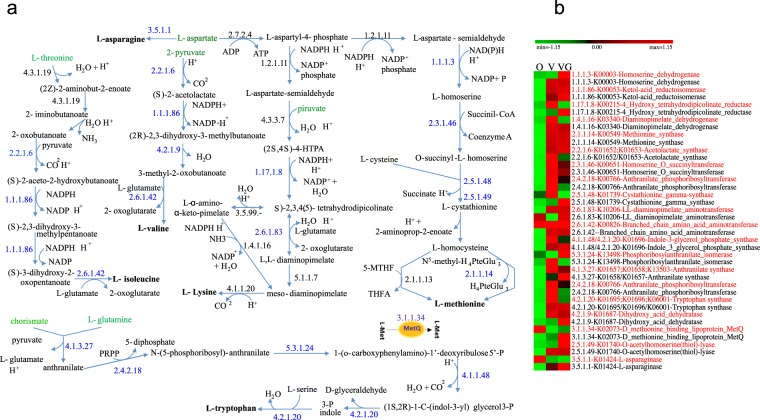
Reconstruction of the microbial pathways in the intestine involved in the biosynthesis of L-methionine, L-lysine, L-isoleucine, L-valine and L-tryptophan using DESeq statistically significant differences for genes and proteins identified from multi-omics data sets belonging to omnivores (O), vegans (V) and vegetarians (VG). Panel a, schematic representation of metabolic pathways for the biosynthesis of amino acids. The blue numbers indicate enzymes that were differentially (FDR < 0.05) detected among diet groups. Principal metabolites are colored in green. Panel b, heatmap showing the differentially detected genes (red characters) and proteins (black characters) in the diet groups. The colors of the scale bar denote gene and protein abundance (indicated in blue characters in panel a), with 1.15 indicating the highest abundance (red) and −1.15 indicating the lowest abundance (green) between diet groups.

The metabolism of nitrogen relates to both proteolytic system and metabolism of amino acids, assuming a pivotal role for microbial growth and survival. The abundance of several peptidases (amidohydrolases, protease 4, M16 and M23 family peptidases, peptidase E and T, and methionine aminopeptidase) was higher (p-values generated using DESeq2, by applying a Wald test followed by Benjamini–Hochberg correction) in vegans and/or vegetarians (Supplementary Table S5) with respect to omnivores. No statistically significant differences occurred among dietary habits by using DNA-seq data and looking for gene levels for the same enzymes. Meta-genomic and -proteomic data reconstructed complete biosynthetic pathways for isoleucine (Ile) from threonine; L-lysine (Lys) and L-methionine (Met) from L-aspartate; L-valine (Val) from pyruvate; and L-tryptophan (Trp) from chorismate and L-glutamine (Fig. 4a). The level of several enzymes was lower in omnivores with respect to vegans and/or vegetarians (Fig. 4b). The relative amount of the specific methionine transport system (MetQ, E.C. 3.1.1.34) was the highest in vegetarians. Besides, the relative abundance of enzymes (e.g., E.C. 4.1.1.48; E.C. 4.2.1.20) responsible for Trp biosynthesis mainly associated to vegans and vegetarians. Compared to omnivores, vegans and, especially, vegetarians had a higher level of L-asparaginase (E.C. 3.5.1.1, K01424), which converts L-asparagine into L-aspartate.

### Diet modulates the capacity of the intestinal microbiome to synthesize vitamins and cofactors

**Figure 5:**
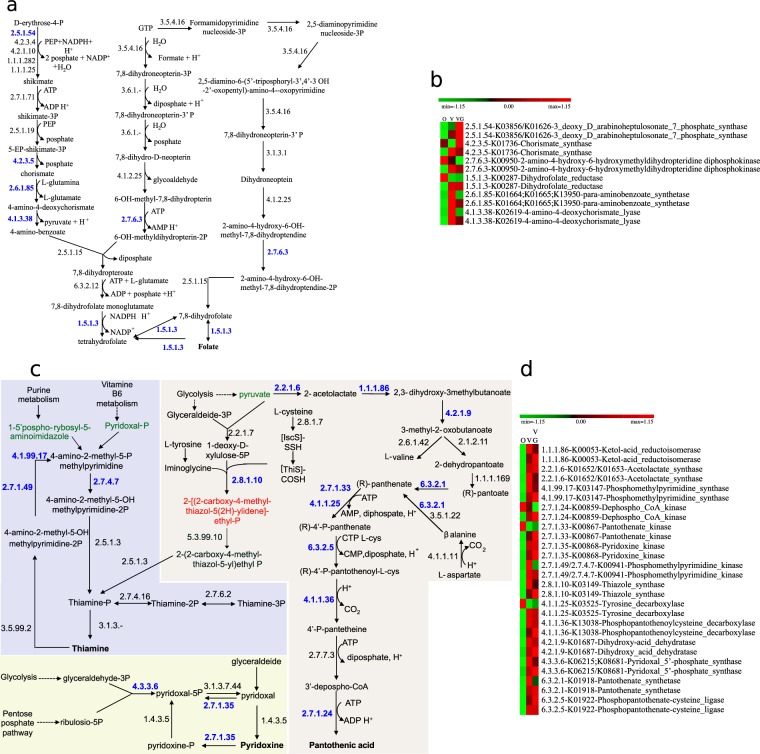
Reconstruction of the microbial intestinal pathways involved in the biosynthesis of folate (Panels a, b) and thiamine, pyridoxal, pantothenic acid and coenzyme A (Panels c, d) statistically significant differences for genes and proteins identified from multi-omics data sets by applying the DESeq2 R package and belonging to omnivores (O), vegans (V) and vegetarians (VG). Panels a and c, schematic representations of metabolic pathways for the biosynthesis of folate and thiamine, pyridoxal, pantothenic acid and coenzyme A. The blue numbers indicate enzymes that were differentially (FDR < 0.05) detected among diet groups by applying the DESeq2 package. Panels b and d, heatmap showing the differentially detected genes (red characters) and proteins (black characters) in the diet groups. The colors of the scale bar denote gene and protein abundance (indicated in blue characters in panel a), with 1.15 indicating the highest abundance (red) and −1.15 indicating the lowest abundance (green) between diet groups.

Meta-genomic and -proteomic data revealed the presence of enzymes responsible for the *de novo* synthesis of folate (Fig. 5a). Most of these significantly different enzymes (p-values generated using DESeq2, by applying a Wald test followed by Benjamini–Hochberg correction) were at higher levels in vegans and/or vegetarians with respect to omnivores (Fig. 5b). Biosynthetic genes for menaquinols (menaquinol-6 to menaquinol-13) were present in all the meta-genomes, but menaquinol-6 and −9 were mainly detectable in the vegan and vegetarian meta-proteomes. All meta-genomes and -proteomes shared genes and proteins for the biosynthesis of deoxyxylulose-5P (DXP) from pyruvate, glyceraldehyde-3P, L-cysteine or L-tyrosine. DXP is required for the synthesis of thiamine and pyridoxal (Fig. 5c). In particular, the level of *thiG*, encoding the key enzyme thiazole synthase (E.C. 2.8.1.10), was the lowest in omnivores (Fig. 5d). Indeed, the biosynthesis of thiamine from pyridoxal-P or 1-(5’-phospho-ribosyl)−5-aminoimidazole primarily related to vegans and vegetarians. The biosynthesis of pantothenic acid (vitamin B5) and CoA, starting from pyruvate and L-aspartate, was part of the core meta-genomes and -proteomes. Nevertheless, the higher relative abundance of proteins responsible for the biosynthesis of pantothenic acid and CoA was mostly associated to vegans and vegetarians. Besides, the higher abundance of proteins responsible for the biosynthesis of pyridoxal and pyridoxine also mostly related to the same dietary habits.

### Correlations between meta-proteome and diet

We further correlated meta-proteomic data to dietary habits. The R package psych was used to compute Pairwise Spearman’s correlations; adjusted p-values were generated by applying the Bonferroni correction (Supplementary Table S6). Fibers positively correlated with flagellin (R = 0.646, FDR = 0.003). The key enzyme phosphate acetyltransferase positively correlated with daily fiber (R = 0.617, FDR = 0.033) and legume (R = 0.698, FDR = 0.043) intakes, and negatively (FDR < 0.05) correlated with meat, preserved meat and fish intakes. Other enzymes responsible for propanoate and butanoate metabolisms (e.g., malate L-lactate dehydrogenase) positively correlated (FDR < 0.05) with daily intakes of fiber, vegetable oil, vegetable proteins and other components of the vegetable-based diet. On the contrary, the above enzymes negatively correlated with the intake of animal products.

### Anti-proliferative effects of the intestinal microbiome in the HT29 human colon carcinoma cell line

**Figure 6:**
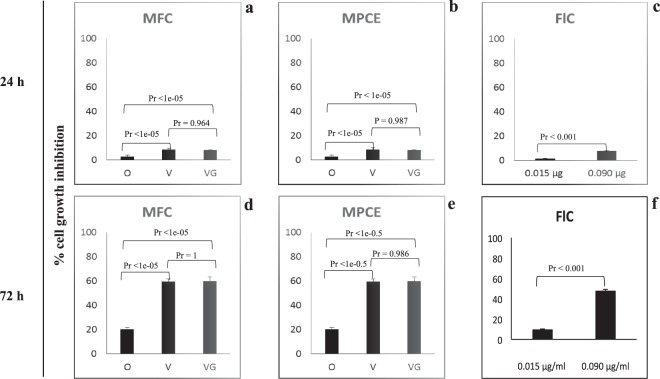
The intestinal microbiota inhibits the proliferation of colon cancer cells. The capacity of microbial fecal cells (MFCs) extracted from 22 omnivores (O), 20 vegetarians (VG) and 19 vegans (V) and the corresponding microbial protein cell extracts (MPCEs) and flagellin (FlC) at two different concentrations (0.015 µg/ml of DMEM for O and 0.090 µg/ml for VG and V MPCE samples) to affect cell viability was assessed by the sulforhodamine B assay at different time-points in human HT29 colon cancer cells. Percentages of growth inhibition for colon cancer cells treated with MFCs, MPCEs or FlC compared to control (cells treated with PBS only), presented as the mean value ± s.d. from three replicates for each of the 61 samples. All data shown are representative of three independent experiments using fecal samples collected at three time-points in one month. Corrected P-values were obtained by using ANOVA test and Tukey test for multiple test correction.

The effects of SCFAs and flagellin on growth of the human HT29 adenocarcinoma cell line, together with their tumor-suppression and anti-inflammatory activities have been extensively studied in literature^22,23^. We here analysed HT29 colon carcinoma cell line in 61 volunteers in order to study the effect of flagellin on their viability. Microbiomes were from 22 omnivorous, 20 vegetarian and 19 vegan fecal samples (27 analyzed in this study plus 34 additional samples belonging to a previous larger cohort^14^). Microbial fecal cells (MFCs) (ca. 8 log cells/ml of Dulbecco’s modified Eagle’s medium [DMEM]) and the corresponding microbial protein cell extracts (MPCEs) (15 mg/ml) were used to perform growth inhibition assays. The ANOVA results (adjusted p-values corrected for multiple tests by applying the Tukey test) showed how growth of HT-29 cells was significantly inhibited by MFC and MPCE treatments (Fig. 6a,b,d,e). Hence, the strongest inhibition (*P* < 1e-05) was after 72 h and, especially, using MFCs from vegans and vegetarians. The highest ability of vegans and vegetarians to inhibit the HT29 cell growth could be due to the flagellin concentrations. As estimated by nanoHPLC coupled to nanoESI-MS/SM, the lower value of flagellin was found in MFCs of omnivores (ca. 0.001 µg/mg) than vegan and vegetarians (ca. 0.006 µg/mg) (*P* < 1e-05) MFCs from vegetarians *vs*. omnivores, *P* < 1e-05 MFCs from vegans *vs*. omnivores and *P* = 0.964 MFCs from vegetarians *vs*. vegans. To prove the effect of flagellin on the viability of HT29 cells, a pure preparation of flagellin was also tested at the same concentration found in 15 mg of MPCE from omnivores (e.g., 0.015 µg/ml of DMEM) or vegans and vegetarians (0.090 µg/ml). Flagellin inhibited the viability of HT29 cells in a dose- and time-dependent manner (Fig. 6c,f, Supplementary Table S7). Compared to control, the strongest inhibition by flagellin (47%, *P* < 0.001) was after 72 h (Fig. 6f).

### The intestinal microbiome increases the expression of interleukins, Toll-like receptor 5 (TLR-5) and the lectin RegIIIα

**Figure 7:**
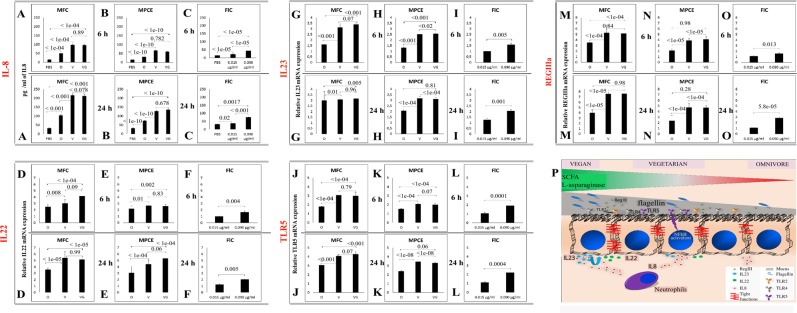
The intestinal microbiota induces the expression of interleukins, Toll-like receptor 5 and the lectin RegIIIα. The capacity of microbial fecal cells (MFCs) extracted from 22 omnivores (O), 20 vegetarians (VG) and 19 vegans (V) and the corresponding microbial protein cell extracts (MPCEs) and flagellin (FlC) at two different concentrations (0.015 µg/ml of DMEM for O and 0.090 µg/ml for VG and V MPCE samples) to affect the expression of IL-8 (panels A–C), IL-22 (D–F) and IL-23 (G–I), Toll-like receptor 5 (J–L) and the lectin RegIIIα (M–O) was assessed at 6 and 24 h in human HT29 colon cancer cells. The levels of expression of each gene in colon cancer cells treated with MFCs, MPCEs or FlC, compared to control (cells treated with PBS only), presented as the mean value ± s.d. from three replicates for each of the 61 samples. All data shown are representative of three independent experiments using fecal samples collected at three time-points in one month. Differences between control and treated cells were considered statistically significant when p < 0.05. A schematic representation of how interleukins, TLR-5 and RegIIIα work synergically is provided in panel P.

We investigated whether MFCs and MPCEs affected the immune response in HT29 cells and in order to disclose significant differences between the samples, we computed an ANOVA test corrected for multiple comparisons by using the Tukey test (see material and method). Compared to PBS-exposed cells, exposure (6 h) of HT29 cells to omnivorous MFCs significantly increased the release of IL-8 (50 ± 2.14 and 15 ± 0.24 pg/ml, respectively, *P* < 1e-04; Fig. 7a, Supplementary Table S8). The release of IL-8 was higher when HT29 cells underwent treatments with vegetarian or vegan MFCs (95.9 ± 4.69 and 97.11 ± 4.87 pg/ml, respectively, *P* < 1e-04) (*P* < 1e-04 for vegetarians *vs*. omnivores, and *P* < 1e-04 for MFCs from vegans *vs*. omnivores). A prolonged exposure (24 h) to MFCs increased IL-8, but the trend did not vary.

To focus on the effect of microbial proteins without metabolite (e.g., butyrate) interferences, HT29 cells underwent exposure to MPCEs. As expected, the effect of MPCEs was lower than that of MFCs (*P* < 0.05; Figs. 7a,b, Supplementary Table S8). Compared to omnivorous MPCEs, exposure to vegetarian MPCEs increased the release of IL-8 (Fig. 7b, Supplementary Table S8). Vegan MPCEs behaved similarly. After 6 h of exposure, the release of IL-8 was similar between treatment with 0.090 µg/ml of flagellin and vegetarian or vegan MPCEs (Fig. 7c, Supplementary Table S8). At 24 h, the efficiency of flagellin was lower than MPCEs but significantly (*P* < 1e-05) higher than PBS.

Exposure of HT29 cells to MFCs, MPCEs or flagellin increased the expression of the interleukins IL-22 (Fig. 7d–f) and IL-23 (Fig. 7g–i, Supplementary Table S8) and TLR-5 (Fig. 7j–l, Supplementary Table S8). Vegetarian and vegan MFCs or MPCEs promoted the highest (*P* < 0.05) induction of interleukins and TLR-5. Compared to PBS-exposed cells, exposure (6 h) of HT29 cells to omnivorous MFCs significantly increased the expression of the bactericidal C-type lectin RegIIIα (fold change, 3.50 ± 0.05, *P* < 1e-04). The expression of RegIIIα was the highest with vegetarian or vegan MFCs, both after 6 and 24 h (Fig. 7m, Supplementary Table S8). Compared to PBS-exposed cells, the exposure of HT29 cells to 0.090 µg/ml of flagellin increased the expression of RegIIIα (Fig. 7o, Supplementary Table S8). Figure 7p reports a schematic representation of the synergistic activity of interleukins, TLR-5 and RegIIIα.

## Discussion

Using a non-redundant catalogue of intestinal bacterial genes and proteins, we assessed how the intestinal microbiome responds and adapts to different dietary habits. Highlighting the relationships between diet and gut microbiome, and their repercussions in microbiome-host metabolic and immune interactions is one of the main current challenges in microbiology^24–26^. According to our data, the phylogenetic composition was not useful in discriminating omnivorous, vegetarian and vegan dietary habits. As previously reported^2,11^, the inter-individual variability was high. Few phylogenetic traits were associated to vegan and vegetarian (*Lachnospira*) or omnivorous (*Ruminococcaceae*) diets. Nevertheless, higher diversity at sub-genus level may exist as we recently showed for *Prevotella* and *Bacteroides*^27,28^. As previously described^2^, Gene Ontology (GO) categories based on meta-genome catalogue of the intestinal microbiome were shared in healthy individuals. However, vegans and vegetarians were associated with the highest abundance of genes responsible for flagellar assembly, chemotaxis and two-component systems. Vegans and vegetarians also harbored functional potential, related to carbohydrate, amino acid, cofactor and vitamin metabolisms. The meta-proteomic approach completed the estimation of the microbiome metabolic adaptation to dietary habits. The relationships between DNA and protein content in the complex microbiome ecology are poorly understood. Gene copy number and protein abundance evaluation is a critical step useful to evaluate how changes in DNA relate to changes in proteins. Some published data suggest that changes in the metagenome reflect changes in the metaproteome. We observed how, compared to meta-genomic, a higher variability of the GO categories occurred at proteomic level. Although the sample size in this study is relatively small, our results may reflect the environmental adaptation and meta-genomic plasticity of the intestinal microbiome under different dietary conditions. Vegetarians showed the highest relative amount of proteins for many GO categories. Proteins responsible for flagellar assembly (e.g., flagellin) were overrepresented in vegetarians and vegans. These findings, along with boosted chemotaxis functions, indicated that microbiomes of individuals with plant-based diet, which is rich of non-digestible carbohydrates, might had developed strategies to get physical access to nutrients. Overall, flagella correlated with cell adhesion and biofilm formation, and represented virulence factors that increase the host immune response^29^.

The intestinal microbiome showed numerous genes that are responsible for carbohydrate transport and metabolism. Proton-coupled active transport components (MFS, GPH, and the ATP binding cassette superfamily) and group trans-locators (PTS-GFL superfamily) were the most frequently identified. Depending on the type of available carbohydrate, bacteria modulate the synthesis of specific transporters. Compared to omnivorous, vegan and, especially, vegetarian fecal samples showed a higher relative number of carbohydrate-hydrolyzing enzymes and transport systems. This feature enhanced the carbohydrate metabolism, positively affected the levels of acetate, butyrate and propionate, which regulate glucose and energy homeostasis^30^, and maintain the integrity of the intestinal epithelial barrier^31,32^. The synthesis of propionate proceeds via succinic acid, with methyl-malonyl-CoA decarboxylase as the key enzyme^33^. Based on the core genes and proteins that we detected, methyl-malonyl-CoA decarboxylase is the main biosynthetic enzyme for propionate in human intestinal microbiomes. This pathway is connected, by pyruvate, to both C6 and C5 compounds. Interestingly, vegan and vegetarian diets induced some enzymes (e.g., malate-L-lactate dehydrogenase), which were positively correlated to vegetable-based foods. The acetyl-CoA-utilizing pathway is the main route to synthesize butyrate, followed by lysine, glutarate and 4-aminobutyrate^34^. Here, we show that vegan and vegetarian diets activated the super-pathway of *Clostridium* acidogenic fermentation, which produces butyrate and acetate from pyruvate/acetyl-CoA. This explains the strong correlation between fiber intake and fecal concentration of butyrate^14,35^ and strengthens the intake of such nutrients as pivotal for health-promoting metabolism. Acetyl-CoA and lysine pathways were detectable in the same fecal samples, suggesting the adaptation to a protein-rich diet^34^. Our data confirmed that alternative pathways (lysine and glutarate) for butyrate biosynthesis^36^ were present and mainly associated to omnivorous diet.

Vegans and even more vegetarians showed higher levels of protein-hydrolyzing enzymes than omnivores. This finding correlates to the high resistance of vegetable proteins to human-gut digestion and to the higher intake of legumes, as recorded in vegan and vegetarian questionnaires. The biosynthetic pathways for biogenic amines in vegans and vegetarians did not differ from those of the omnivores. The same was detectable for enzymes catalyzing the conversion of carnitine, choline or betaine into trimethylamine. This result was consistent with the absence of trimethylamine and TMAO in the fecal samples. Consequently, the high intake of carnitine, choline or betaine by omnivores with low adherence to MD might be responsible for the high level of TMAO detected in their urines^14,15^. We showed active biosynthetic pathways for Met, Lys, Ile, Val and Tyr, which represents an interesting feature of the functional meta-genome, since humans lack this biosynthetic ability. Biosynthetic pathways for folate, thiamine, pantothenic acid, pyridoxal and pyridoxine were also active. Several enzymes catalyzing these pathways had higher levels in vegans and/or vegetarians with respect to omnivores.

Overall, we found that omnivorous, vegetarian or vegan diets had an impact on the microbial synthesis of proteins (e.g., flagellin and L-asparaginase) and metabolites (e.g., butyrate and pyridoxal/pyridoxine) that are linked to mechanisms of oncogenesis and tumor suppression^37^. The microbial fecal cells, their protein extracts and flagellin showed anti-proliferative effects towards HT29 colon carcinoma cells. Although our model is oversimplified, it is worth noting that the strongest inhibitory effect was found by using high concentrations of flagellin and, especially, the intestinal microbiome from vegetarians and vegans. Previously published data demonstrated that HT29 cells express TLR-5^38^ and that some probiotic bacteria inhibit cell growth and viability of colon carcinoma cells. Binding to TLR-5, flagellin induces the IL-22-dependent production of antibacterial lectins of the RegIII family^39^. Commonly, the induction of IL-22 is associated to the production of IL-23 from macrophages/dendritic cells. Nevertheless, colorectal carcinoma cells such as HT29 release IL-23^40^. Flagellin has attracted pharmacological interest to develop vaccines^41^ and because its capability to stimulate immune responses^42^. Thus, we hypothesized the existence of additional protection against gut-infecting bacteria and pathogens in vegans and vegetarians. Flagellin enhances tumor-specific CD8 + T cell immune responses^43^ and improves the defense towards genital cancer in mice^44^. A protective effect of flagellin against other types of cancer, as well as the capability to increase tumor necrosis, was described in animal models^45^. Currently, pathogen inhibition and SCFA production are the main colon cancer-preventing effects of fiber and vegetable-enriched diets. By correlating the fiber daily intake and the level of flagellin in fecal samples, we hypothesized a tangible role of fiber-rich diets in protecting against tumor occurrence. Nevertheless, flagellin alone showed weaker inductive effects on IL-8, IL-22, IL-23, TLR-5 and RegIIIα than vegetarian or vegan MPCEs, which indicated also a presumptive role of other proteins. We noticed that fiber- and vegetable-enriched diets exhibited higher levels of enzymes involved in tumor suppression (L-asparaginase and ribonuclease)^46^.

In conclusion, our multi-omics approach shed new light on complex diet-microbiome interactions. Intestinal microbial metabolism consists of many enzymatic reactions that function together in a synchronized manner. Complex regulatory mechanisms maintain the functional balance among individual pathways. Although the need of further investigations, our data demonstrated how responses from the intestinal microbiome to vegetable-rich diets primarily include: an increased cell motility to access nutrients, an increased catalytic activities for carbohydrates and food proteins, the synthesis/release of bioactive metabolites/proteins and potentially beneficial impacts on human health.

## Methods

### Participant recruitment

Volunteers were recruited through advertisements and using flyers, which were distributed in the areas surrounding 4 different Italian cities: Bologna, Parma, Torino and Bari. Thirty healthy adult volunteers (15 men and 15 women) were enrolled, with an age of 25–55 years (36 ± 7.0), and a BMI > 18 (21.89 ± 2.20). The volunteers had been following an omnivorous, an ovo-lacto-vegetarian or a vegan diet for at least one year. Omnivorous, vegetarian, or vegan diets were validated by one week FFQ (Food Frequency Questionnaire)^47^. The sample set included individuals who followed an omnivorous (total no = 10; 5 men and 5 women), an ovo-lacto-vegetarian (total no = 10; 4 men and 6 women) or a vegan (total no = 10; 5 men and 5 women) diet. Volunteer features, recruitment and exclusion criteria, dietary information, sample collection and storage procedures are reported in De Filippis *et al*., 2016^14^. Prospective participants were excluded according to the following criteria: V, VG and O regime followed for less than one year, age under 18 or over 60, regular consumption of drug, regular supplementation with prebiotics or probiotics, consumption of antibiotics in the previous 3 months, evidence of intestinal pathologies (Crohn’s disease, chronic ulcerative colitis, bacterial overgrowth syndrome, constipation, celiac disease, Irritable Bowel Syndrome) and other pathologies (type I or type II diabetes, cardiovascular or cerebrovascular diseases, cancer, neurodegenerative diseases, rheumatoid arthritis, allergies), pregnancy and lactation. All participants were asked questions about consumption of animal product in order to understand if their dietary habits in the last year diverged from the self-declared diet type. The subjects were instructed on how to self-collect the samples; all materials were provided in a sterile convenient, refrigerated, specimen collection kit (VWR, Milan, Italy). Faecal samples were collected on the same day of three consecutive weeks, and the three samples were pooled before microbiome, metaproteome and metabolome analyses. Home collected samples were transferred to the sterile sampling containers using a polypropylene spoon and immediately stored at 4 °C by the volunteers. The specimens were transported to the laboratory within 12 hours of collection at a refrigerated temperature. Containers were immediately stored at −80 °C. Food and beverage intake was estimated by means of a 7-day weighed food diary, which was completed every day for a total of one week, to collect metadata and to confirm the type of diet. The intake of macronutrients and micronutrients was calculated using the Microsoft Access application coupled to the European Institute of Oncology food database (European Institute of Oncology, 2008).

### Ethical statement

The study protocol was approved by the Ethics Committee of: (a) Azienda Sanitaria Locale (Bari) (protocol N.1050), (b) Azienda Ospedaliera Universitaria of Bologna (protocol N.0018396), (c) Province of Parma (protocol N.22884) and (d) University of Torino (protocol N.1/2013/C) after having ascertained its compliance with the dictates of the Declaration of Helsinki (IV adaptation). All methods were performed in accordance with relevant guidelines and regulations. All patients provided written informed consent prior to participation in the study protocol. The study protocol was registered on ClinicalTrials.gov, with the identified number NCT02118857.

### DNA extraction and sequencing

Triplicate fecal aliquots collected from each volunteer were pooled for DNA extraction. Ten grams of the pooled sample was aseptically homogenized with 90 ml of Ringer’s solution (Oxoid) for 2 min in a Stomacher. A 2-ml aliquot was collected and centrifuged at the maximum speed for 30 s; the supernatant was removed, and the DNA was extracted from the pellet using a Powersoil DNA kit (MO-BIO, Carlsbad, CA, USA) according to the manufacturer’s instructions. Single-end DNA library construction (one 151-bp) was performed by using the TruSeq DNA library preparation kit, and shotgun sequencing for the HiSeq. 1500 platform (Illumina, San Diego, CA, USA) was performed according to the manufacturer’s instructions (G4L Company, Salerno, Italy).

### Functional meta-genomic annotation and statistical analysis

Raw sequencing reads were quality-trimmed (Phred score < 30), and reads shorter than 60 bp were discarded using the SolexaQA +  + (v3.1.7.1) software^48^. The remaining reads were aligned against the Integrated Gene Catalogue^17^ (IGC) of human gut developed within the MetaHit project using Bowtie2 (v2.3.5.1) software^49^ with the following parameters: -t -f -D 20; -R 3; -N 0; -L 20; -i S, 1, 0.50 – local. Reads that showed the best hit ( > 90% of identity over at least 30% of the query length) against the IGC were extracted using SAMtools (version 1.9) and normalized to the total read number mapped to the whole catalogue. An average value of 90% of reads were mapped against the IGC and only genes with KEGG ID were extracted and further used for downstream analysis (3,644 KEGG Orthology (KO) genes). Shotgun reads were also assembled with Velvet v1.2.10 with default parameters^50^. Reads that are human contaminants have been discarded by using the BMTagger software (ftp://ftp.ncbi.nlm.nih.gov/pub/agarwala/bmtagger/). Each contig was analyzed by using the automated gene prediction and annotation pipeline PROKKA^51^ v1.12. In order to reconstruct metabolic pathways the FASTA and genbank files relative to the set of annotated contigs were parsed then used as input for Pathway Tools v19.0.

Data normalization and the determination of differentially abundant genes, among the three dietary groups, were then conducted using the Bioconductor DESeq2 package^20^ in the statistical environment R with default parameters. P values were adjusted for multiple testing using the Benjamini-Hochberg procedure, which assesses the FDR.

PCoA was performed with R “adegenet” package (https://cran.r-project.org/web/packages/adegenet/adegenet.pdf) using the gene relative abundance based on Euclidean, Bray-Curtis and Jaccard distances. The Random Forests algorithm was used to discriminate genes among diet groups. The phylogenetic characterization of the shotgun sequences was assessed using MetaPhlAn2^18^ software with default parameters. The resulting biological observation matrix (.biom files) was then imported into QIIME^52^ to produce an OTU table at the genus level. In order to find differences in microbiome composition among the samples as a function of diet the Wilcoxon test in R was used.

### Alpha diversity indices were estimated by the R phyloseq package

Spearman’s non-parametric correlations through the psych package of R were used to study the relationships between the relative abundance of microbial taxa abundance and dietary variables. The correlation plots were visualized in R using the made4 package of R.

A succinct step-by-step workflow summarizes the analyses carried out both for the meta-genomic and the meta-proteomic counterparts (Supplementary Fig. S3).

### Protein extraction, denaturation, digestion and desalting

Pooled fecal samples (2 g) were suspended in 18 ml of ice-cold Tris-buffered saline (TBS) buffer and homogenized using a lab Stomacher. The homogenate was passed through a 20-μm vacuum filter unit to remove larger fibrous material and human cells and centrifuged (1000 rpm for 5 min) to pellet bacterial cells. The pellet was collected, washed with 5 ml of Tris-HCl (50 mM pH 7.5) to remove attached human proteins and lysed via sonication. Proteins were precipitated with 20% TCA, digested by trypsin and analyzed by UHPLC-MS/MS.

Samples were prepared for digestion using the filter-assisted sample preparation (FASP) method^53^. Briefly, the samples were suspended in 1% SDC, 50 mM Tris-HCl, pH 7.6, and 3 mM DTT, sonicated briefly, and incubated in a Thermo-Mixer at 40 °C, 1000 rpm for 20 min. The samples were clarified by centrifugation, and the supernatant was transferred to a 30 kDa MWCO device (Millipore) and centrifuged at 13 kg for 30 min. The remaining sample was buffer exchanged with 1% SDC, 100 mM Tris-HCl, pH 7.6, then alkylated with 15 mM iodoacetamide. The SDC concentration was reduced to 0.1%. The samples were digested using trypsin at an enzyme-to-substrate ratio of 1:100 overnight at 37 °C in a Thermo-Mixer at 1000 rpm. Digested peptides were collected by centrifugation. A portion of the digested peptides, approximately 20 µg, were desalted using reversed phase stop-and-go extraction (STAGE) tips^54^. Peptides were eluted with 80% acetonitrile and 0.5% acetic acid and lyophilized to near dryness in a SpeedVac (Thermo Savant) for approximately 1 h.

### Liquid chromatography-tandem mass spectrometry

Each digestion mixture was analyzed by liquid chromatography (LC) by using an Easy-nLC 1000 UHPLC system (Thermo Fisher). Mobile phase A was 97.5% MilliQ water, 2% acetonitrile, and 0.5% acetic acid. Mobile phase B was 99.5% acetonitrile and 0.5% acetic acid. The 240-min LC gradient ran from 0% B to 35% B over 210 min and then to 80% B for the remaining 30 min. Samples were loaded directly into the column. The column was 50 cm × 75 μm I.D. and packed with 2 micron C18 media (Thermo Easy Spray PepMap). The LC was interfaced to a quadrupole-Orbitrap mass spectrometer (Q-Exactive, Thermo Fisher) via nanoelectrospray ionization using a source with an integrated column heater (Thermo Easy Spray source). The column was heated to 50 °C. An electrospray voltage of 2.2 kV was applied. The mass spectrometer was programmed to acquire tandem mass spectra from the top 10 ions in the full scan from 400 to 1200 m/z by data-dependent acquisition. Dynamic exclusion was set to 15 s, singly charged ions were excluded, the isolation width was set to 1.6 Da, the full MS resolution was set to 70,000 and the MS/MS resolution was set to 17,500. Normalized collision energy was set to 25, max fill MS was set to 20 ms, max fill MS/MS was set to 60 ms and the underfill ratio was set to 0.1%. The mass spectrometer RAW data files were converted to mzML format using msconvert.

### Functional meta-proteomic annotation and statistical analysis

Mascot Generic Format (MGF) files were generated from mzML using the Peak Picker HiRes tool, part of the OpenMS framework. All search instances were performed on an Amazon Web Services–based cluster through the Proteome Cluster interface. Proteome Cluster builds monthly species- and genus-specific protein sequence libraries from the most current UniProtKB distribution. The most recent protein sequence libraries available from UniProtKB were used. MGF files were searched using X!Tandem^55^, both with the native^56^ and k-score^57^ algorithms and using OMSSA^58^. XML output files were parsed and non-redundant protein sets were determined using the Proteome Cluster based on previously published rules^59^. MS1-based isotopic features were detected, and peptide peak areas were calculated using the Feature Finder Centroid tool from the OpenMS framework^60^. Data normalization and the determination of differentially abundant proteins, among the three dietary groups, were conducted using the Bioconductor DESeq2 package^20^ in the statistical environment R with default parameters. Wald test p-values were corrected for multiple testing by using the Benjamini-Hochberg post hoc procedure.

Looking for evidence of structure among the analysed diet groups, we filtered out non-informative non-core proteins, i.e. those proteins that occurred with a maximum of 15% in each diet group. We ran DAPC by using the adegenet R package. In this multivariate analysis we used the belonging to the sample diet group as the *a priori* clustering condition and we retained 4 principal components. We plotted the first two discriminant functions with the scatter function of the adegenet R package. In order to ascertain if DAPC classification is consistent with the original clusters (known from diet diaries), we used the “assignplot” R function to calculate the proportions of successful reassignments (based on the discriminant functions). This function is particularly useful when prior biological groups are used, as one may infer admixed or misclassified individuals.

### Microbiome pathway reconstruction

PT software version 19.0^21^ and the coupled MetaCyc multiorganism database were used to reconstruct metabolic pathways. For the meta-genomic counterpart, the assembled genbank and .fasta files were both used to generate the .pt (pathologic) format. For the proteomic data batch, the protein output was converted into the .pf format, miming the genbank entry fields. The .pf and .pt supported formats were then used, through the built-in Pathologic software, to obtain new PGDB databases.

The numbers of reactions (total reactions in the base pathways) and pathways (base pathways) where compared in each sample and used to generate 0/1 matrices. The PT software allowed us to infer the prediction of metabolic pathway hole in our meta-genomic and -proteomic samples. The REST-style version of the KEGG API utility (http://www.kegg.jp/kegg/rest/) was used to enrich the protein dataset in terms of KEGG codes and EC numbers.

### Gas-chromatography mass spectrometry/solid-phase microextraction (GC-MS/SPME) analysis

According to the manufacturer’s instructions, the DVB/CAR/PDMS fibre (Supelco, Bellefonte, PA, USA) was exposed to headspace for 40 min to extract volatile organic compounds (VOCs) from fecal samples. VOCs were thermally desorbed by immediately transferring the fiber into the heated injection port (220 °C) of a Clarus 680 (Perkin Elmer, Beaconsfield UK) gas chromatography equipped with an Rtx-WAX column (30 m × 0.25 mm i.d., 0.25 μm film thickness) (Restek) and coupled to a Clarus SQ8MS (Perkin Elmer) with source and transfer line temperatures kept at 250 and 210 °C, respectively. Each chromatogram was analyzed for peak identification using the National Institute of Standard and Technology 2008 (NIST) library. Quantitative data of the identified compounds were obtained by interpolation of the relative areas versus the internal standard area.

### Fecal microbes and preparation of protein extracts for HT29 cell line assays

Thirty fecal samples analyzed by a multi-omic approach, plus 31 additional samples belonging to the previous larger cohort (13), for a total of 22 omnivores, 20 vegetarians and 19 vegans, were used. MFCs and MPCEs were obtained using the protocols applied for meta-proteomic analysis. MFC samples were washed with sterile PBS and added to DMEM at a final cell density (O.D. 620 nm) of 0.65 UA, corresponding to ca. 8 log cells/ml. MPCEs were analyzed by the Bradford method to quantify the total protein concentrations. The flagellin content in the 61 MPCE samples was also purified by liquid chromatography and quantified by nano-HPLC coupled with nano-ESI-MS/MS. Each MPCE was added to DMEM at a final protein concentration of 15 mg/ml. Flagellin was also used at final concentrations of 0.015 and 0.090 µg/ml in DMEM.

### Cell line

Based on the above results showing that diet modulates the microbial synthesis of molecules/proteins (e.g., SCFA and flagellin) involved in oncogenesis or tumor suppression, the microbiomes of 61 volunteers were tested using the human HT29 colon carcinoma cell line. HT29 cells were cultured in DMEM containing fetal bovine serum (10%, FBS, Life Technologies), 2 mM glutamine and 100 u/ml penicillin/100 μg/ml streptomycin (Life Technologies) at 37 °C in the presence of 5% CO_2._ For the co-incubation experiments with MFCs, MPCEs, fecal microbiomes or flagellin (InvivoGen, San Diego, CA, USA), the cells were maintained at 37 °C under CO_2_-independent conditions and cultured with the above-described standard DMEM supplemented with 25 mM HEPES.

### HT29 cell viability assays

The cell viability of HT29 cells was assessed by the SRB assay^61^ using an initial cell density of 5,000 or 20,000 cells/well, respectively. The cells were incubated with MFCs, MPCEs or flagellin for 24, 48 and 72 h. After washing with PBS, the cells were fixed with 10% TCA. Staining of cells was performed using SRB for 30 min, and the cells were flushed repeatedly with 1% acetic acid. SRB was desorbed using 10 mM Trizma, and the plate was read at 492 nm using a microplate reader. Cells incubated in DMEM alone were used as controls.

### Gene expression analyses of HT29 cells

HT29 cells grown with DMEM or DMEM plus MFCs, MPCEs or commercial flagellin for 6 and 24 h were washed twice with PBS containing Pen-Strep and 50 µg/ml gentamicin and stored at −80 °C until use. Total RNA was extracted from the HT29 cells using a commercial kit (Ribospin Minikit-GeneAll, Seoul, Korea). cDNA was synthesized from 2 μg of template RNA in a 20-μl reaction volume using the High-Capacity cDNA Reverse Transcription Kit (Applied Biosystems, Monza, Italy). Ten microliters of total RNA was added to the Master Mix and subjected to RT-PCR in a thermal cycler (Stratagene Mx3000P Real Time PCR System, Agilent Technologies Italia S.p.A., Milan, Italy). The cDNA was amplified and detected through Taqman primer-probe sets (Applied Biosystems) (IL8, Hs00174103_m1; IL22, Hs01574154_m1; IL23A, Hs00372324_m1; TLR5, Hs01920773_s1; and REG3A, Hs00170171_m1). Human glyceraldehyde-3-phosphate dehydrogenase (GAPDH) was used as the housekeeping gene and detected through Taqman primer-probe Hs999999_m1. The relative fold change in expression was normalized to GAPDH expression. All procedures were performed according to the manufacturer’s instructions. TLR-5 was quantified by chromatin immunoprecipitation (ChIP) using EZChIPTM chromatin immunoprecipitation kit (Upstate) as described by Kumar Thakur *et al*.^62^. In details, HT29 cell pellets were resuspended in the lysis buffer of the kit, and the chromatin was precipitated overnight with 2 μg of rabbit antibodies against RNA polymerase II, Sp1, Sp3, acetyl-H3, acetyl-H4, p300, HDAC1 or IgG (negative control). At the end of incubation, samples were treated with Protein G agarose for 1 h. The immunoprecipitated complex was washed and subsequently extracted with elution buffer. DNA-protein complexes were reversed and DNA was purified by ethanol precipitation. The relative binding of proteins to the TLR-5 promoter was quantitatively analyzed by qPCR.

### Enzyme-linked immunosorbent assay (ELISA)

Cell culture supernatants were analyzed for IL-8, IL-22 and IL-23 release in triplicate using an ELISA kit (Human IL-8/CXCL8; IL-22 and IL-23 DuoSet ELISA R&D Systems, Minneapolis, MN, USA CN: DY208, DY782 and DY1290 respectively).

### Statistical analysis

All data coming from gas-chromatography mass spectrometry-solid-phase microextraction (GC-MS/SPME) were obtained at least in triplicates. The GC-MS/SPME analysis, was carried out on transformed data followed by separation of means with Tukey’s HSD, using a statistical software Statistica for Windows (Statistica 6.0 per Windows 1998, (StatSoft, Vigonza, Italia).

For cell line assay statistical analyses (data at least in triplicate), differences between groups were analyzed using the ANOVA test. The correction for multiple comparisons was performed using the Tukey test and the function glht (general linear hypothesis tests) in “multcomp” R package^63^.

Degree of association between genera and nutrients were assessed by Spearman correlation coefficients than clustered by Euclidean distance and Ward linkage hierarchical clustering. Correlations between enzymes abundances and dietary intake were assessed by using Spearman’s correlation coefficients (FDR < 0.05 and R > 0.6); the p-values were corrected for multiple testing by using the Bonferroni adjustment within the psych R package.

## Supplementary information

Supplementary info.

Supplementary Table S1.

Supplementary Table S2.

Supplementary Table S3.

Supplementary Table S4.

Supplementary Table S5.

Supplementary Table S6.

Supplementary Table S7.

Supplementary Table S8.

## Data Availability

Sequences filtered for human reads and trimmed of low-quality bases have been uploaded to the National Center for Biotechnology Information Sequence Read Archive (NCBI SRA; SRP083099, Bioproject ID PRJNA340216).
