# Supplementary material for: Diet influences the functions of the human intestinal microbiome
Source: Sci Rep. 2020 Mar 6;10:4247. doi: 10.1038/s41598-020-61192-y (PMC7060259; doi:10.1038/s41598-020-61192-y)
Supplement: Supplementary file 1 — Supplementary info. [file 41598_2020_61192_MOESM1_ESM.pdf]

Supplementary Information for:

Diet influences the functions of the human intestinal microbiome

Maria De Angelis[1], Ilario Ferrocino[2], Francesco Maria Calabrese[1,3], Francesca De Filippis[4], Noemi Cavallo[1], Sonya Siragusa[1], Simone Rampelli[5], Raffaella Di Cagno[6], Kalliopi Rantsiou[2], Lucia Vannini[7], Nicoletta Pellegrini[8], Camilla Lazzi[8], Silvia Turroni[5], Nicola Lorusso[3], Mario Ventura[3], Marcello Chieppa[9], Erasmo Neviani[8], Patrizia Brigidi[5], Paul W. O'Toole[10], Danilo Ercolini[4], Marco Gobbetti[6*], Luca Cocolin[2]

[1]Department of Soil, Plant and Food Science, University of Bari Aldo Moro, Bari, Italy.
[2]Department of Agricultural, Forest and Food Science, University of Turin, Grugliasco, Italy.
[3]Department of Biology, University of Bari Aldo Moro, Bari, Italy.
[4]Department of Agricultural Sciences and Task Force on Microbiome Studies, University of Naples Federico II, Portici, Italy.
[5]Department of Pharmacy and Biotechnology, Alma Mater Studiorum University of Bologna, Bologna, Italy.
[6]Faculty of Science and Technology, Free University of Bozen, Italy.
[7]Department of Agricultural and Food Sciences, Alma Mater Studiorum University of Bologna, and Inter-Departmental Centre for Industrial Agri-Food Research, Alma Mater Studiorum University of Bologna, Cesena, Italy.
[8]Food and Drug Department, University of Parma, Parma, Italy.
[9]National Institute of Gastroenterology "S. de Bellis", Castellana Grotte, Bari, Italy.
[10]Department of Microbiology and Alimentary Pharmabiotic Centre, University College Cork, Cork, Ireland.

* Prof. Marco Gobbetti, Faculty of Science and Technology, Free University of Bozen, Italy, E-mail address: marco.gobbetti@unibz.it Tel.: +39 0471 017215.

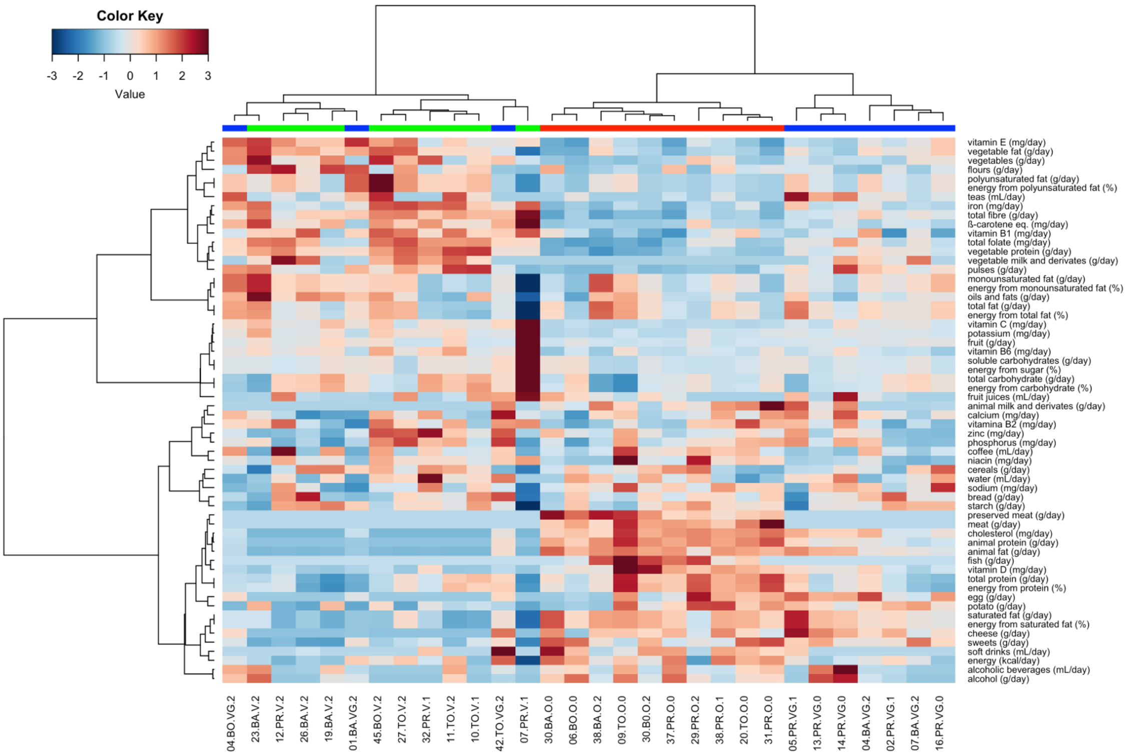

Supplementary Figure S1. Average-linkage clustering based on the Spearman distance of omnivores (O), vegans (V) and vegetarians (VG) and dietary information. The columns are color-coded as follows: red, O; green, V; and blue, VG. Rows and columns are clustered by Ward linkage hierarchical clustering. The intensity of the colors, denoted as the Z-score, represents the degree of correlation between samples and dietary information, as measured by Spearman's correlations; red indicates a high correlation, and blue indicates a low correlation. Sample codes include (i) the type of diet as O, omnivorous; V, vegan; or VG, vegetarian; and (ii) Mediterranean diet adherence as low, 0; medium, 1; or high, 2.

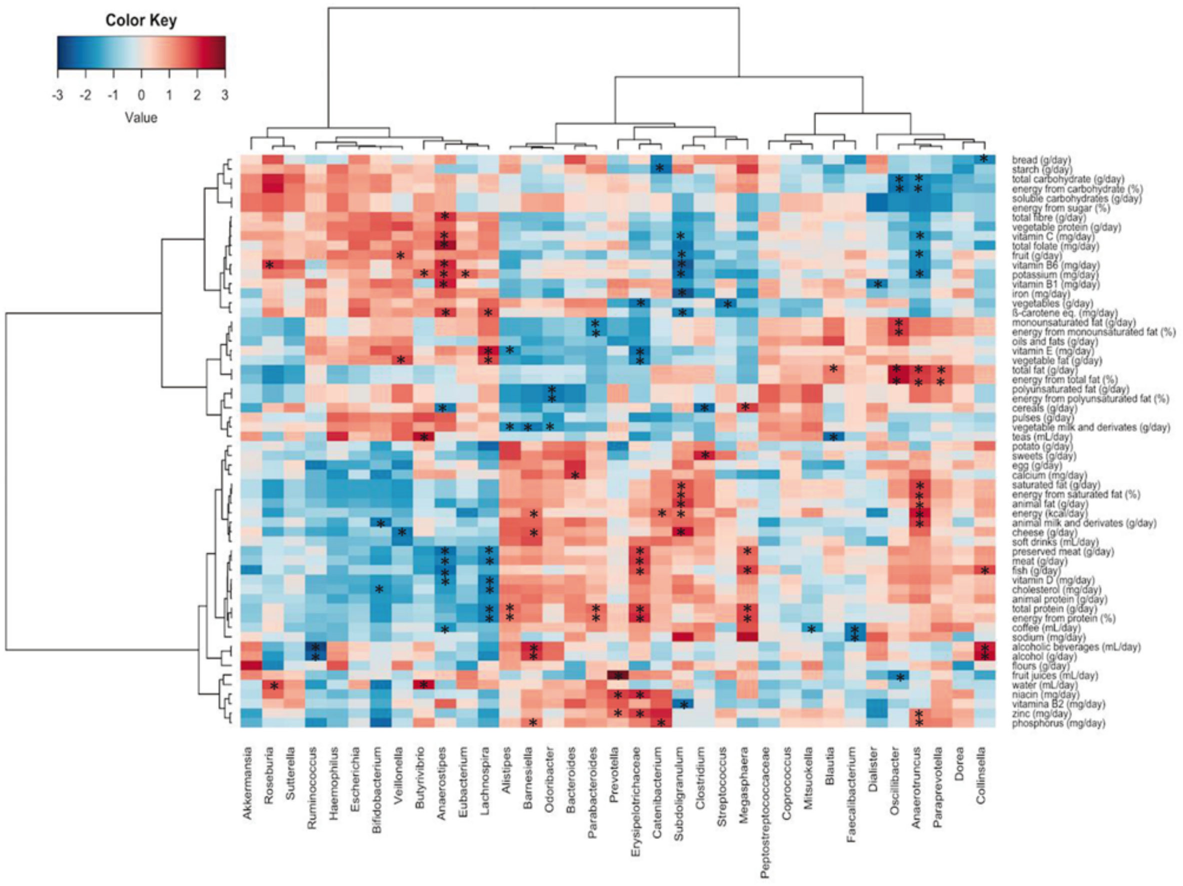

Supplementary Figure S2. Correlation between dietary information and fecal microbiome composition. Heatplot showing Spearman's correlations between microbial genera and dietary information. Rows and columns are clustered by Euclidean distance and Ward linkage hierarchical clustering. The intensity of colors represents the degree of association between genera and nutrients, as measured by Spearman's correlations. Asterisks indicate significant correlations (FDR < 0.05).

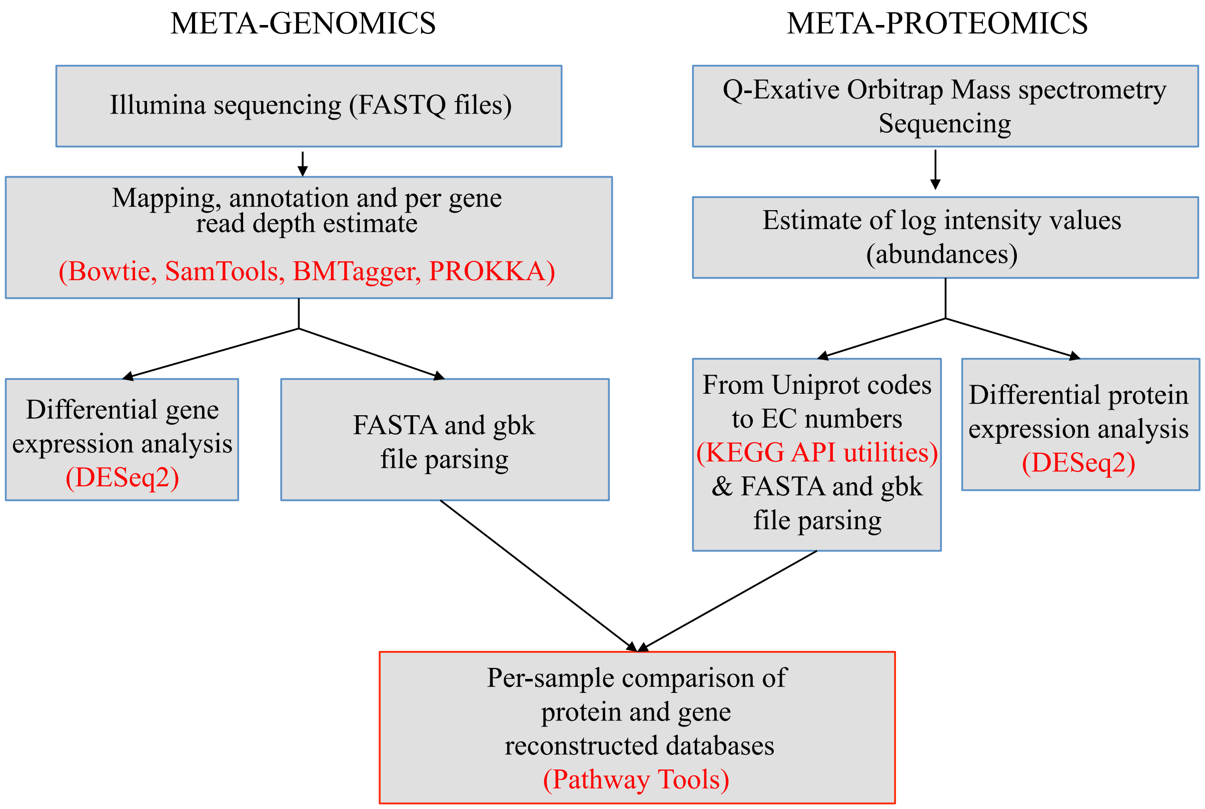

Supplementary Figure S3. Display of features of followed analysis workflow for meta-genomic and meta-proteomic data. Illumina and Mass spectrometry sequencing raw data files were analysed thanks to the software reported in red. Gene and protein annotation files converged in the Pathway Tools metabolic map reconstruction.
